# Vitamin D deficiency is associated with higher disease activity and the risk for uveitis in juvenile idiopathic arthritis - data from a German inception cohort

**DOI:** 10.1186/s13075-018-1765-y

**Published:** 2018-12-13

**Authors:** Claudia Sengler, Julian Zink, Jens Klotsche, Martina Niewerth, Ina Liedmann, Gerd Horneff, Christoph Kessel, Gerd Ganser, Angelika Thon, Johannes-Peter Haas, Anton Hospach, Frank Weller-Heinemann, Arnd Heiligenhaus, Dirk Foell, Angela Zink, Kirsten Minden

**Affiliations:** 1 German Rheumatism Research Center, a Leibniz Institute, Charitéplatz 1, 10117 Berlin, Germany; 2 Institute for Social Medicine, Epidemiology and Health Economics, Charité Universitätsmedizin Berlin, Berlin, Germany; 3 Center for General Pediatrics and Neonatology, Asklepios Klinik Sankt Augustin, Sankt Augustin, Germany; 4 University hospital Cologne, Cologne, Germany; 5 Department of Pediatric Rheumatology and Immunology, University of Münster, Münster, Germany; 6 Clinic of Pediatric Rheumatology, St. Josef-Stift Hospital, Sendenhorst, Germany; 7 Department of Pediatric Pneumology, Allergology and Neonatology, Children’s Hospital, Medical School, Hanover, Germany; 8 German Center for Pediatric and Adolescent Rheumatology, Garmisch-Partenkirchen, Germany; 9 Olgahospital Kinderklinik, Stuttgart, Germany; 10 Prof.-Hess-Kinderklinik, Bremen, Germany; 11 Department of Ophthalmology and Ophtha-Lab at St. Franziskus Hospital, Muenster, Germany; 12 University of Duisburg-Essen, Essen, Germany; 13 Department of Rheumatology and Clinical Immunology, Charité Universitätsmedizin Berlin, Berlin, Germany

**Keywords:** Vitamin D, Juvenile idiopathic arthritis, Disease activity, Uveitis

## Abstract

**Objective:**

The objective was to evaluate the 25(OH) vitamin D (25(OH)D) status of patients with juvenile idiopathic arthritis (JIA) and determine whether the 25(OH)D level is associated with disease activity and the course of JIA.

**Methods:**

Patients ≤ 16 years of age with recently diagnosed JIA (< 12 months) were enrolled in the inception cohort of patients with newly diagnosed JIA (ICON), an ongoing prospective observational, controlled multicenter study started in 2010. Clinical and laboratory parameters were ascertained quarterly during the first year and half-yearly thereafter.

Of the 954 enrolled patients, 360 patients with two blood samples taken during the first 2 years after inclusion and with follow up of 3 years were selected. The serum 25(OH)D levels were determined and compared with those of subjects from the general population after matching for age, sex, migration status and the month of blood-drawing.

**Results:**

Nearly half of the patients had a deficient 25(OH)D level (< 20 ng/ml) in the first serum sample and a quarter had a deficient level in both samples. Disease activity and the risk of developing JIA-associated uveitis were inversely correlated with the 25(OH)D level (β = − 0.20, 95% CI − 0.37; 0.03, hazard ratio 0.95, 95% CI 0.91; 0.99, respectively).

**Conclusion:**

In this study, 25(OH)D deficiency was common and associated with higher disease activity and risk of developing JIA-associated uveitis. Further studies are needed to substantiate these results and determine whether correcting 25(OH)D deficiency is beneficial in JIA.

## Key messages

- Vitamin D deficiency was common, as it was found in 44% of patients with juvenile idiopathic arthritis (JIA) and even in 62% of healthy peers
- Low levels of 25(OH)D in patients with JIA were associated with higher disease activity and higher risk of developing JIA-associated uveitis

## Introduction

Over the last decade, it has become clear that vitamin D is more than the “bone vitamin” that regulates calcium homeostasis and bone mineralization. In addition to its pleiotropic functions in different cells and tissues, the differentiation, polarization and activity of immune cells are affected by 1,25 (OH)_2_ vitamin D3 [1]. This is the biologically active form that results from the double hydroxylation of cholecalciferol that is ingested or produced in the skin by ultraviolet sunlight. Several studies have shown an association between low vitamin D, mostly measured as 25(OH)vitamin D3 (25(OH)D), and higher incidence and severity of autoimmune disorders, such as type 1 diabetes mellitus, chronic inflammatory bowel disease, rheumatoid arthritis (RA) and multiple sclerosis [1]. Nisar et al. conducted a meta-analysis of several studies evaluating patients with JIA with reported serum vitamin D levels [2]; however, the parameters that were measured (25(OH)D, 1,25(OH)_2_D3), clinical outcomes and the cutoff values that were used differed substantially. Currently, the measurement of 25(OH)D is considered standard because it is stable and reflects the vitamin D provision of the last weeks to months. In our study, we wanted to (1) evaluate the 25(OH)D level in patients with newly diagnosed JIA and compare it with that in individuals from the general population; (2) analyze whether disease activity is correlated with the 25(OH)D level and (3) determine whether the 25(OH)D level might predict the disease course.

## Methods

### Patients

The inception cohort of newly diagnosed patients with JIA (ICON) has been described in detail elsewhere [3]. Briefly, patients diagnosed with JIA according to the International League of Associations for Rheumatology (ILAR) criteria [4] over the previous 12 months were included in this prospective, multicenter, observational cohort study. On the basis of standardized questionnaires and pediatric rheumatologic assessments, clinical and demographic data and information on medication use were collected every 3 months during the first year and every 6 months thereafter. Vitamin D supplementation was not ascertained. The active joint count, the physician’s global assessment of disease activity on a numeric rating scale (NRS, 0–10, 0 = best), the parents’ global assessment of wellbeing (NRS) and the antinuclear antibody (ANA) status (negative/positive based on the standards of the local laboratory that carried out this assay) were recorded. A slit lamp examination was performed by an ophthalmologist every 3–6 months according to the screening guidelines by the German study group “Uveitis in childhood” [5], and the findings were documented on a specific uveitis documentation form. Patients 8 years of age and older and their parents provided informed assent/consent for participation. The study was approved by the ethics committee of the Charité – Universitätsmedizin Berlin.

### Serum samples

Serum samples were taken as part of routine laboratory controls in ICON and were collected, frozen and stored at the ICON biobank at the University of Münster, Germany, until they were shipped to Berlin, Germany, to perform the 25(OH)D assay for all samples at the same time at a medical laboratory. For our analysis, we selected patients for whom a pair of serum samples was available. The first sample was collected at a visit that occurred between baseline and the 9-month follow up (94% at baseline or at 3-month follow up), and the second sample was collected at a visit that occurred between the 3-month follow up and the 36-month follow up (92% until the 1-year follow up), with a mean interval of 7 months (SD 5) between blood draws. The time points for serum collection in male and female subjects were equally distributed in winter (October–March) and summer (April–September).

### 25(OH) vitamin D measurement and cutoff values

The LIAISON 25 OH Vitamin D TOTAL Assay (direct competitive chemiluminescence immunoassay, DiaSorin Inc., Stillwater, MN, USA) was used to quantitatively determine the 25(OH)D level in the serum samples. The measurement range of this assay ranges from 4 ng/ml (10 nmol/l) to 150 ng/ml (375 nmol/l). To compare the 25(OH)D levels of patients with healthy subjects, we used data from the German National Health Interview and Examination Survey for Children and Adolescents (KIGGS), which was conducted from May 2003 to May 2006, to obtain representative data on health status and selected laboratory values of children and adolescents aged 0–17 years across Germany (for details, please refer to [6]). Valid measurements of serum 25(OH)D, also performed with the LIAISON 25 OH Vitamin D TOTAL Assay, from 10,015 participants of this study were available from public-use files. Control subjects were matched with a ratio 1: 1 for age, sex, month of blood draw and migration background. The latter was defined as “mother’s and/or father’s country of origin not Germany”. This variable integrates several factors like skin pigmentation but also cultural habits like type of clothing and veiling that possibly influence the vitamin D production in the skin and thereby the 25(OH)D serum level [7].

According to the data from several studies that included clinical (e.g., fracture risk) and laboratory parameters (e.g., calcium uptake), it has been proposed vitamin D deficiency as 25(OH)D < 20 ng/ml (< 50 nmol/l) [8–10]. Using the negative relationship between the serum 25(OH)D and parathyroid hormone (PTH), it was shown that PTH began to increase at 25(OH)D of 75 (30 ng/ml) or 78 nmol/l (31.2 ng/ml) [11, 12]. Holick et al. determined the following categories that integrate both cutoff values that we used for our analysis: 25(OH)D deficiency, < 20 ng/ml (< 50 nmol/l); 25(OH)D insufficiency, 20–29 ng/ml and 25(OH)D sufficiency, ≥ 30 ng/ml [13]. A 25(OH)D level < 20 ng/ml or ≥ 30 ng/ml in both of the patient’s serum samples was defined as stable deficient or stable sufficient vitamin D status, respectively, implicating prolonged deficiency or sufficiency of vitamin D.

### Outcome and statistics

The disease activity of patients with JIA was measured by the clinical Juvenile Arthritis Disease Activity Score (cJADAS-10), a composite score (0–30, 0 = best) based on the physician’s global assessment of disease activity, the parents’ global assessments of wellbeing, and the number of active joints up to a maximum of 10, according to McErlane et al. [14]. For the present analysis, (a) the disease activity, (b) the development of the extended form of oligoarthritis and (c) the occurrence of uveitis up to 3 years after inclusion in ICON in relation to the 25(OH)D level were investigated.

Serum 25(OH)D levels were compared by the paired *t* test, and the categories of serum 25(OH)D levels between patients and controls were compared by conditional logistic regression analyses. The association between disease activity and 25(OH)D level was assessed by linear regression analysis. The association between the likelihood of uveitis and serum 25(OH)D, including the covariates of prior methotrexate (MTX) therapy and established uveitis risk factors, such as age at JIA onset, female sex, oligoarticular JIA onset, and ANA positivity, were analyzed using a multivariable Cox proportional hazard model. Multivariable logistic regression analysis was used to investigate the proportion of oligoarthritis patients who progressed to extended oligoarthritis until the 3-year follow up, with serum 25(OH)D as a predictor. *P* values < 0.05 were considered statistically significant. The data were analyzed using SAS software version 9.4 (SAS Institute, In der Neckarhelle 162, 69,118 Heidelberg).

## Results

The subgroup of patients whose 25(OH)D levels were analyzed did not differ from the whole ICON study population in terms of the male-to-female ratio, age at study inclusion or symptom onset and ILAR category (Table 1). Migration background was found in 28.1% of the study population.

**Table 1:** Demographic and clinical data of the ICON study population

	ICON patients with 25(OH)D measurement	All patients of the ICON study
Number	360	954
Female (%)	67.5	67.2
Age at study inclusion, years, mean (SD)	7.1 (4.6)	7.9 (4.8)
Duration between symptom onset and diagnosis, months, median (IQR)	3 (2–7)	3 (1–6)
Duration between diagnosis and ICON enrollment, months, median (IQR)	1.4 (0.5–4.4)	1.5 (0.4–4.7)
Patients		
- on DMARD at inclusion, *n* (%)	178 (49)	471 (49)
- on MTX at inclusion, *n* (%)	164 (46)	441 (46)
- on biologic drug at inclusion, *n* (%)	14 (4)	49 (5)
Median (IQR) duration (months) of		
- DMARD use at inclusion	1.2 (0.5–3.4)	1.2 (0.5–3.5)
- MTX use at inclusion	1.2 (0.5–3.5)	1.2 (0.5–3.4)
- Biologic drug use at inclusion	1.1 (0.4–2.9)	0.9 (0.3–2.9)
ANA positive at study inclusion, *n* (%)	228 (63)	517 (54)
	ILAR category, *n* (%)	ILAR category, *n* (%)
Systemic arthritis	15 (4)	35 (4)
Oligoarthritis	173 (48)	445 (47)
Psoriatic arthritis	18 (5)	39 (4)
Enthesitis-related arthritis	30 (8)	100 (11)
Polyarthritis, rheumatoid factor positive	5 (1)	15 (2)
Polyarthritis, rheumatoid factor negative	96 (27)	252 (26)
Other arthritis	23 (6)	68 (7)

*ICON* Inception cohort of newly diagnosed patients with juvenile idiopathic arthritis, *25(OH)D*: 25(OH) vitamin D, *SD* standard deviation, *IQR* interquartile range, *DMARD* disease-modifying antirheumatic drug, *MTX* methotrexate, *ANA* antinuclear antibody, *ILAR* International League of Associations for Rheumatology

The mean 25(OH)D levels and the distribution of deficient and sufficient 25(OH)D levels in the study population and control subjects are summarized in Table 2. Mean 25(OH)D (first serum sample) in patients was insufficient and was slightly, although significantly, higher than that from matched subjects from KIGGS (22.8 ng/ml (SD 10.3) and 19.6 ng/ml (SD 11.2), respectively, *p* < 0.001). Nearly half of the patients (44%) had a deficient 25(OH)D level in the first serum sample.

**Table 2:** Comparison of 25(OH)D levels in patients from ICON and subjects from the KIGGS study and number/proportion of patients with a stable deficient or stable sufficient 25(OH)D status

	ICON patients, first sample			KIGGS subjects		
	25(OH)D ng/ml mean (SD)	25(OH)D deficient (< 20 ng/ml) Number (%)	25(OH)D sufficient (≥ 30 ng/ml) Number (%)	25(OH)D ng/ml mean (SD)	25(OH)D deficient (< 20 ng/ml) Number (%)	25(OH)D sufficient (≥ 30 ng/ml) Number (%)
All (*n* = 360)	22.8 (10)	158 (44)	75 (21)	19.6 (11)	221 (62)	57 (16)
Male (*n* = 117)	22.7 (10)	54 (49)	26 (24)	18.5 (11)	68 (62)	14 (13)
Female (*n* = 243)	22.8 (10)	104 (42)	49 (20)	20.1 (11)	153 (62)	43 (17)
	ICON patients, both samples					
	25(OH)D ng/ml mean (SD)	Stable 25(OH)D deficient (2 < 20 ng/ml) Number (%)	Stable 25(OH)D sufficient (2 ≥ 30 ng/ml) Number (%)			
All (*n* = 360)	22.1 (8)	87 (25)	23 (7)			
Male (*n* = 117)	22.2 (8)	29 (25)	8 (7)			
Female (*n* = 243)	22.1 (8)	58 (24)	15 (6)			
ILAR categories						
Systemic JIA (*n* = 15)	21.8 (12)	5 (33)	2 (13)			
Oligoarthritis (*n* = 173)	23.3 (8)	29 (17)	14 (8)			
Polyarthritis, rheumatoid factor negative (*n* = 96)	20.9 (8)	32 (33)	4 (4)			
Polyarthritis, rheumatoid factor positive (*n* = 5)	23.7 (9)	1 (20)	1 (20)			
Enthesitis-related arthritis (*n* = 30)	21.1 (6)	8 (27)	1 (3)			
Psoriatic arthritis (*n* = 18)	22.4 (7)	6 (33)	0 (0)			
Other arthritis (*n* = 23)	20.3 (8)	8 (35)	2 (9)			
Patients with uveitis (*n* = 61)	20.9 (7)	17 (28)	2 (3)			
Patients without uveitis (*n* = 299)	22.4 (8)	72 (24)	22 (7)			
ANA positive patients (*n* = 228)	22.4 (8)	54 (24)	14 (6)			
ANA negative patients (*n* = 120)	21.6 (8)	32 (27)	9 (8)			

### 25(OH)D level, disease activity and therapy

**Fig. 1:**
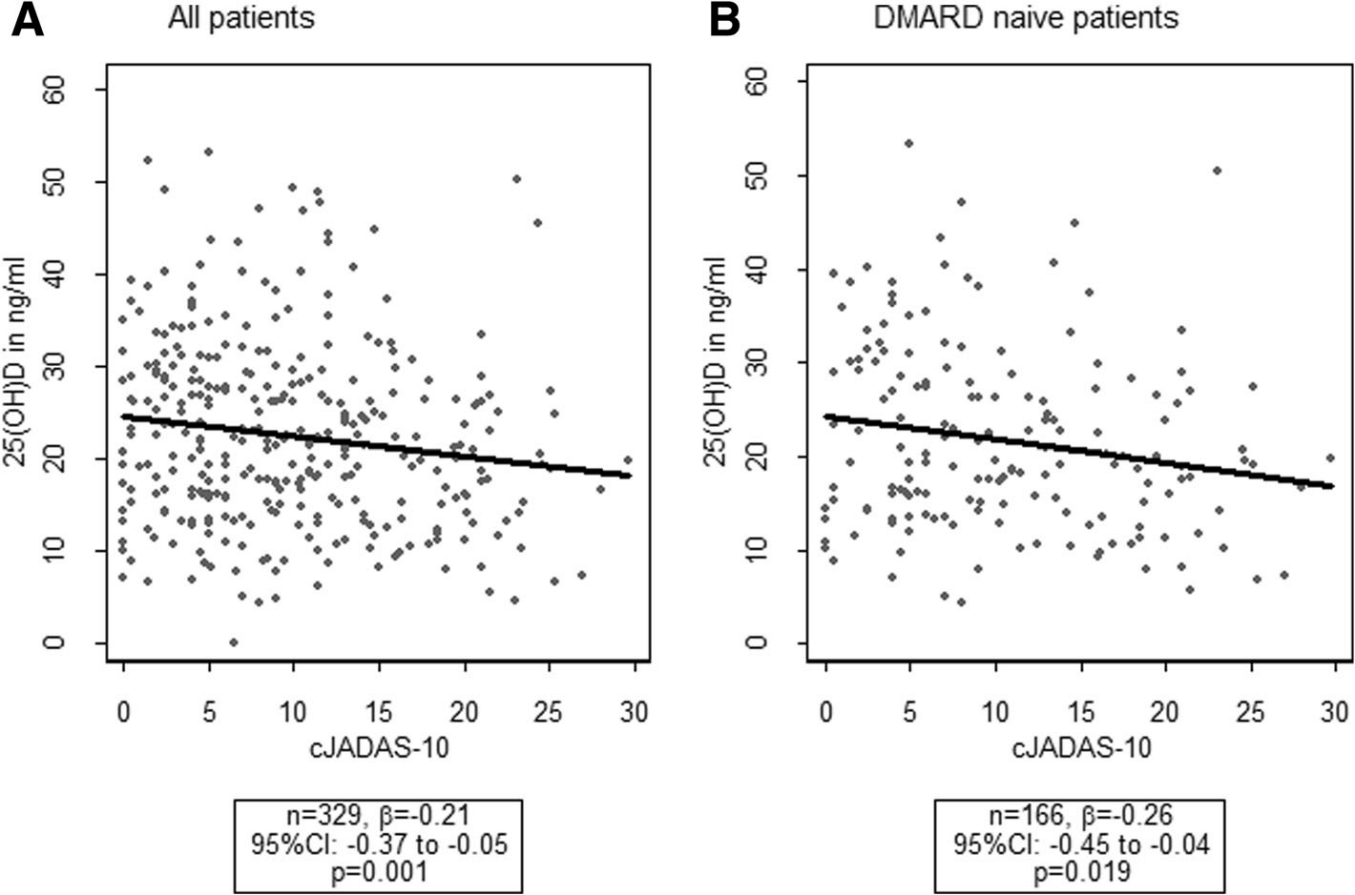
Association between Clinical Juvenile Arthritis Disease Activity Score (cJADAS-10) and 25-hydroxy-vitamin D (25(OH)D) level at first measurement. DMARD, disease-modifying anti-rheumatic drug

We hypothesized that an effect of the vitamin D status later in the disease course might be abrogated by already established therapy. Thus, we used the 25(OH)D level from the first measured sample to analyze possible correlation with disease activity because at this time point, the patients were in an early phase of disease (median disease duration at that time 6.6 (IQR 3.8–11.4) months) and any treatment was initiated only briefly (212 (59%) treated with disease modifying anti-rheumatic drugs (DMARDs), 195 (54%) with MTX, and 18 (5%) with biologic disease-modifying anti-rheumatic drugs (bDMARDs); median therapy duration 1.0 (IQR 0.5–3.5) month). We identified negative correlation between disease activity and the 25(OH)D level (Fig. 1a), which became more pronounced when we analyzed DMARD-naïve patients (Fig. 1b) and only patients with polyarthritis (*n* = 71, β = − 0.38; 95% CI − 0.65; − 0.11; *p* = 0.007).

While in patients receiving (*n* = 208) and not receiving (*n* = 152) conventional synthetic disease modifying anti-rheumatic drugs (csDMARDs) at the first measurement, the mean 25(OH)D level did not differ significantly (22.2 ng/ml, SD 10.1 and 23.8 ng/ml, SD 10.5, respectively), patients receiving systemic glucocorticoid therapy ≥ 0.2 mg/kg body weight prednisolone equivalent (*n* = 19) had significantly lower mean 25(OH)D than patients (*n* = 275) without such systemic glucocorticoid doses (16.4 ng/ml, SD 9.4 and 23.0 ng/ml, SD 10.1, respectively, *p* = 0.014). Patients with 25(OH)D < 20 ng/ml in the first measurement received systemic glucocorticoids ≥ 0.2 mg/kg body weight prednisolone equivalent (*n* = 12, 7.6%) and csDMARDs (*n* = 102, 64.2%) a little more frequently at this time than patients with 25(OH)D > 30 ng/ml (*n* = 2, 2.6% and *n* = 44, 56.4%, respectively); these results were not statistically significant.

### 25(OH)D deficiency in the ILAR categories

Mean 25(OH)D of the two serum samples from each patient was insufficient (22.1 ng/ml (SD 7.8)), with no differences between the sexes. Between the individual ILAR categories, mean 25(OH)D did not vary significantly, while the highest percentage of stable 25(OH)D deficiency was in patients with systemic, psoriatic and rheumatoid factor (RF)-negative polyarticular JIA (Table 2).

### Risk of developing extended oligoarthritis or uveitis

**Fig. 2:**
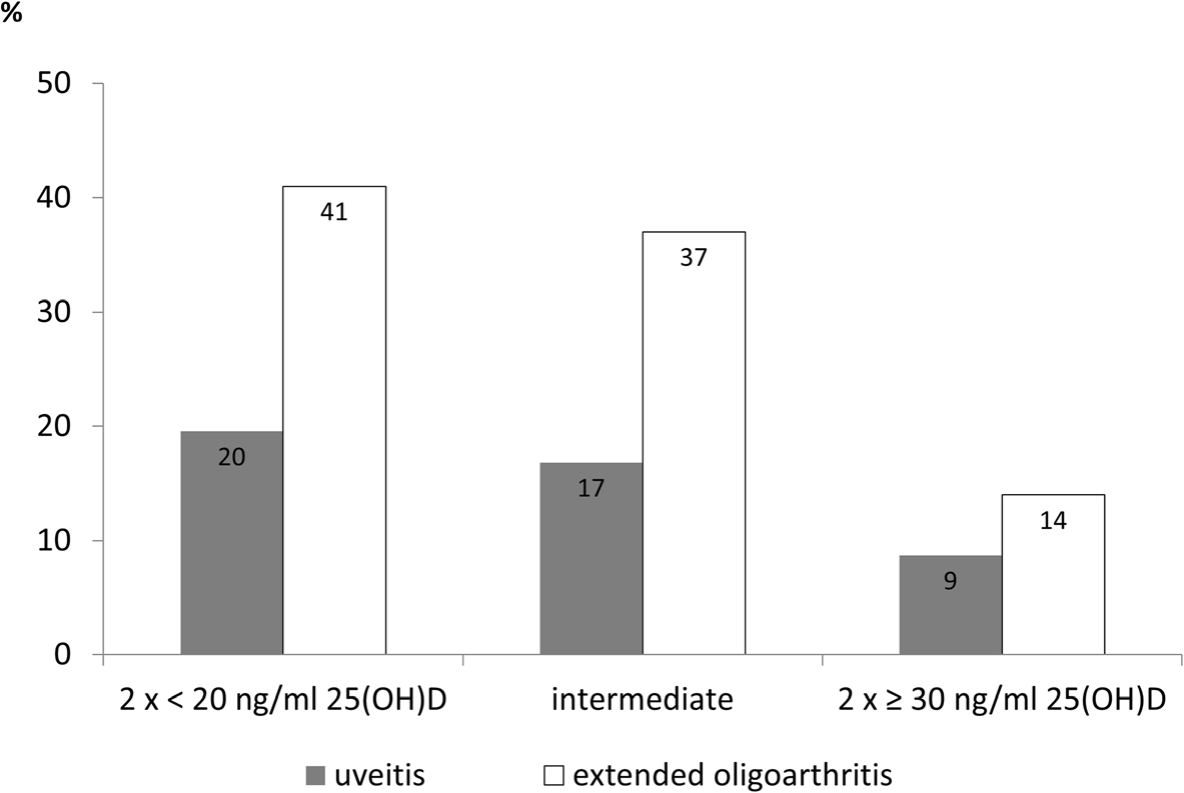
Proportion of patients with stable deficient or stable sufficient 25-hydroxy-vitamin D (25(OH)D) status who developed uveitis or the extended form of oligoarthritis up to the 3-year follow up

We compared patients with a stable deficient 25(OH)D level to those with a stable sufficient 25(OH)D level in terms of the development of the extended form of oligoarthritis or the occurrence of uveitis. Overall, 62 of the 173 patients (36%) with oligoarthritis developed an extended disease course until the 3-year follow up; 18 of these patients already had extended oligoarthritis at the time of enrollment. Twelve of the 29 patients (41%) with oligoarthritis and a stable deficient 25(OH)D level and 2 of the 14 patients (14%) with oligoarticular disease and a stable sufficient 25(OH)D level developed the extended form of disease in the first 3 years in ICON (*p* = 0.034, Fig. 2). This result was not corroborated by the multivariable analysis.

Altogether, 61 of the 360 patients (17%) developed uveitis by the 3-year follow up: 10 of these patients developed uveitis before enrollment and 51 patients developed uveitis during the first 3 years in ICON. The majority had oligoarticular disease (54% persistent/13% extended), followed by RF-polyarthritis (21%). Of the 87 patients with a stable deficient 25(OH)D level, 17 (20%) developed uveitis, whereas only 2 of the 23 patients (9%) with a stable sufficient 25(OH)D level did so (*p* = 0.189, Fig. 2). In the multivariable regression analysis, we showed that the 25(OH)D level was significantly associated with the risk of developing uveitis (Table 3). For every 1 ng/ml increase in 25(OH)D > 22.1 ng/ml (the mean of the cohort), there was a 5% reduction in the risk of developing uveitis. This correlation persisted when we limited the examination to patients with uveitis who had blood drawn for 25(OH)D determination prior to developing uveitis (HR 0.95, 95% CI 0.92; 1.00, *p* = 0.03).

**Table 3:** Risk of developing uveitis in relation to 25(OH)D level (mean of both measurements) and other parameters

	Patients with uveitis *n* = 61	Patients without uveitis *n* = 299	Hazard ratio	95% CI	*p* value
25(OH)D level, mean (SD), ng/ml	21.0 (6.8)	22.4 (8.0)	0.95	0.91; 0.99	0.022
Female sex, *n* (%)	45 (74)	198 (66)	1.32	2.56; 0.68	0.414
Age at JIA onset, mean (SD), years	4.0 (2.9)	7.8 (4.6)	0.84	0.77; 0.93	0.001
Oligoarticular JIA onset, *n* (%)	41 (67)	132 (44)	1.20	0.60; 2.41	0.601
ANA positivity at study inclusion, *n* (%)	58 (95)	170 (57)	5.07	1.50; 17.15	0.009
cJADAS-10^a^ (range 0–30), mean (SD)	5.9 (3.9)	4.8 (3.2)	1.22	1.14; 1.31	< 0.001
MTX treatment^b^, *n* (%)	31 (51)	235 (79)	0.29	0.15; 0.57	< 0.001

*25(OH)D* 25(OH) vitamin D, *JIA* juvenile idiopathic arthritis, *ANA* antinuclear antibodies, *MT*X methotrexate, *cJADAS* Clinical Juvenile Arthritis Disease Activity Score

^a^cJADAS-10: mean score in patients with uveitis were calculated until occurrence of uveitis, in patients without uveitis mean cJADAS-10 score was calculated until 3-year follow up

^b^MTX: patients with uveitis, ever treated with MTX until occurrence of uveitis; patients without uveitis, ever treated until 3-year follow up

## Discussion

In this study, in which vitamin D level was repeatedly prospectively measured in patients observed as standard, with recent-onset JIA, the following clinically relevant findings were ascertained:

- Vitamin D deficiency was common in patients with JIA and even more common in their peers from the general population
- 25(OH)D deficiency was associated with higher JIA disease activity
- The progression from oligoarthritis to an extended disease course occurred more often in patients with 25(OH)D deficiency than in those with 25(OH)D sufficiency
- The 25(OH)D level was inversely correlated with the risk of developing JIA-associated anterior uveitis

Pelajo et al. also identified a high prevalence of vitamin D deficiency when they measured 25(OH)D in children and adolescents with rheumatologic diseases and in children with non-autoimmune disorders (e.g., noninflammatory disorders, infectious diseases, pain amplification syndrome) as controls [15]. The risk of vitamin D deficiency was significantly higher in patients with autoimmune disorders (OR 2.3) than in patients with non-autoimmune diseases; however, no significant differences in the mean 25(OH)D levels between these two patient groups were observed. Our finding that vitamin D deficiency is even more common in control subjects than in patients with JIA is somehow unexpected and cannot be easily explained because we controlled for the possible influencing factors by matching for age, sex, migration background and even the month of blood sampling.

Possibly patients with JIA are screened more frequently for vitamin D deficiency in the course of clinical routine and might consequently receive vitamin D supplements. Pepmueller et al. assessed parameters of bone mineralization in patients with JIA and also found that mean 25(OH)D in patients (28.2 ng/ml) was significantly higher than in healthy controls (22.0 ng/ml) [16].

We identified significant negative correlation between the 25(OH)D level from the first serum sample and JIA activity, as measured by the cJADAS-10. Pelajo et al. did not find this association in their cross-sectional study of 154 patients with JIA but noted that the included patients had established ongoing disease and that the majority had received some kind of disease-modifying treatment [17]. In a subset analysis of newly diagnosed patients with JIA, they identified negative association between the serum 25(OH)D levels and the JADAS-27, but this result was not statistically significant. In our study, there was an inverse relationship between 25(OH)D and disease activity that was even more pronounced in DMARD-naive subjects. Van Hamburg et al. showed that TNF-alpha blockade alone was insufficient to suppress the production of interleukin (IL)-17A and IL-22, which are two key cytokines involved in the pathophysiologic interaction of T helper 17 (Th17) cells and synovial fibroblasts in RA. Only in combination with 1,25(OH)_2_D_3_ did TNF-alpha blockade synergistically suppress inflammatory mediators in RA synovial fibroblast cocultures [18].

We did not observe significant differences in mean 25(OH)D between the JIA categories, but the proportion of 25(OH)D-deficient patients differed among the subgroups. Similar to the work by Stagi et al. [19], we identified the highest percentage of vitamin D deficiency in patients with systemic and polyarticular JIA. Whether this finding is somehow specific to a particular JIA category or whether it is the result rather than the cause of disease severity cannot be deduced from our data.

Analyzing the disease course and outcomes of JIA, we observed a higher proportion of stable 25(OH)D-deficient patients with oligoarthritis who developed an extended disease course during the first 3 years in ICON than of patients with oligoarthritis and a stable sufficient 25(OH)D level. To our knowledge, this association has not been described before, and it requires further investigation.

One of the most relevant extra-articular manifestations of JIA is anterior uveitis, which is frequently associated with complications and can be accompanied by visual impairment. We confirmed the known risk factors for the development of anterior uveitis in patients with JIA, including ANA positivity, young age at JIA onset and no prior treatment with methotrexate [20]. A new finding in our study was that a higher percentage of patients with JIA with stable deficient 25(OH)D developed uveitis until the 3-year follow up than did patients with a stable sufficient 25(OH)D level. The odds of developing uveitis were 5% lower for every 1 ng/ml increase in 25(OH)D. As far as we know, this result has not been previously described in childhood uveitis and may be of great interest in predicting and possibly preventing JIA-associated uveitis. Grotting et al. compared 100 adult patients with and without non-infectious anterior uveitis and demonstrated that hypovitaminosis D (defined as 25(OH)D < 30 ng/ml) was associated with this disease, and the odds of developing uveitis were 4% lower for every 1 ng/ml increase in 25(OH)D [21]. Mitulescu et al. analyzed the influence of vitamin D in patients with ankylosing spondylitis in terms of the development of acute anterior uveitis (AAU) and showed an association between low 25(OH)D and AAU accompanied by increased IL-8 and serum amyloid A as markers of the inflammatory response [22]. Yi et al. showed that in patients with Vogt-Koyanagi-Harada disease, a type of autoimmune pan-uveitis, 1,25(OH)_2_D_3_ was significantly lower in patients with active disease than in patients with inactive disease and in controls. Moreover, they demonstrated that the incubation of peripheral blood mononuclear cells (PBMC) with 1,25(OH)_2_D_3_ resulted in reduced proliferation of PBMC and reduced production of IFN-ƴ and IL-17A in the presence of anti-CD3 and anti-CD28 [23], suggesting that vitamin D supplementation might be an adjunctive treatment option for patients with this form of uveitis.

As a large amount of in vitro data has shown the anti-inflammatory and antiproliferative effects of vitamin D on immune cells [1], a causal link between low 25(OH)D and autoimmune diseases is plausible. However, low 25(OH)D might also be the result rather than the cause of the underlying inflammation in these diseases because it has been shown that 25(OH)D decreases during the acute-phase response parallel to the increase in C reactive protein after surgery [24]. Therefore, Welsh et al. investigated whether the administration of a TNF-α blocker - and thereby reduction of the inflammatory reaction in patients with RA - changes the 25(OH)VD levels. They observed a significant improvement in the Disease Activity Score in 28 joints (DAS28) and a decrease in erythrocyte sedimentation rate (ESR), while median 25(OH)D remained unchanged after treatment with adalimumab [25].

To date, only a few papers have described the effects of vitamin D supplementation in children with autoimmune diseases. Reed et al. examined the effect of 25(OH)D supplementation at 1–2 μg/kg body weight/day over 1 year in 13 children with active polyarticular JIA and described a significant increase in 25(OH)D, but did not observe change in disease activity with this regimen [26]. By contrast, Lima et al. performed a study in adolescents and young adults with systemic lupus erythematosus and showed that the increase in the 25(OH)D was accompanied by reduced disease activity and fatigue and decreased frequency of anti-double stranded (ds)DNA antibodies after 24 weeks of supplementation with 50,000 IU of cholecalciferol per week [27].

## Conclusion

Our study revealed that vitamin D deficiency is common in patients with JIA, although the prevalence was lower than in matched subjects from the general population. In patients with JIA, vitamin D deficiency is associated with higher disease activity and a higher risk of developing uveitis and possibly the extended form of oligoarthritis. The strength of this study is the prospective controlled design with standardized data ascertainment. A limitation of this study is that the any use of vitamin D supplementation was not recorded. To further clarify if cholecalciferol substitution in order to achieve a sufficient 25(OH)D level might have the potential to prevent the development of uveitis and the extended form of oligoarthritis, these associations must be confirmed by an interventional study.

## Abbreviations

25(OH)D: 25-Hydroxy-vitamin D
AAU: Acute anterior uveitis
ANA: Antinuclear antibodies
bDMARD: Biologic disease-modifying antirheumatic drug
CD: Cluster of differentiation
CI: Confidence interval
cJADAS: Clinical Juvenile Arthritis Disease Activity Score
CRP: C-reactive protein
csDMARD: Conventional synthetic disease-modifying antirheumatic drug
DMARD: Disease-modifying antirheumatic drug
FU: Follow up
ICON: Inception cohort of patients with newly diagnosed juvenile idiopathic arthritis
IFN-ƴ: Interferon gamma
IL: Interleukin
ILAR: International League of Associations for Rheumatology
IQR: Interquartile range
JIA: Juvenile idiopathic arthritis
KIGGS: German National Health Interview and Examination Survey for Children and Adolescents
MTX: Methotrexate
NRS: Numeric rating scale
PBMC: Peripheral blood mononuclear cells
PTH: Parathyroid hormone
RA: Rheumatoid arthritis
RF: Rheumatoid factor
SD: Standard deviation
TNF-α: Tumor necrosis factor alpha
